# Non-iterative learning machine for identifying CoViD19 using chest X-ray images

**DOI:** 10.1038/s41598-022-15268-6

**Published:** 2022-07-13

**Authors:** Sahil Dalal, Virendra P. Vishwakarma, Varsha Sisaudia, Parul Narwal

**Affiliations:** 1 University School of Information, Communication and Technology, Guru Gobind Singh Indraprastha University, Sector 16-C, Dwarka, New Delhi, India; 2 Department of Information Technology, Delhi Technological University, Shahbad Daulatpur, Rohini, New Delhi, India; 3 Department of Biotechnology, Chandigarh University, NH-95, Ludhiana - Chandigarh State Hwy, Punjab, 140413 India

**Keywords:** Biomedical engineering, Computer science

## Abstract

CoViD19 is a novel disease which has created panic worldwide by infecting millions of people around the world. The last significant variant of this virus, called as omicron, contributed to majority of cases in the third wave across globe. Though lesser in severity as compared to its predecessor, the delta variant, this mutation has shown higher communicable rate. This novel virus with symptoms of pneumonia is dangerous as it is communicable and hence, has engulfed entire world in a very short span of time. With the help of machine learning techniques, entire process of detection can be automated so that direct contacts can be avoided. Therefore, in this paper, experimentation is performed on CoViD19 chest X-ray images using higher order statistics with iterative and non-iterative models. Higher order statistics provide a way of analyzing the disturbances in the chest X-ray images. The results obtained are quite good with 96.64% accuracy using a non-iterative model. For fast testing of the patients, non-iterative model is preferred because it has advantage over iterative model in terms of speed. Comparison with some of the available state-of-the-art methods and some iterative methods proves efficacy of the work.

## Introduction

Since December 2019, a novel disease named corona virus disease 2019 (or CoViD19), which originated from Wuhan, China, has been spreading all over the world. According to the World Health Organization (WHO), the disease is named after its causing virus i.e. severe accurate respiratory syndrome coronavirus 2 (SARS-CoV-2)^1^. Till date, more than 529.17 million number of cases have been already reported worldwide with 6,304,145 deaths. Maximum cases have been reported in the countries like USA, India, Brazil, and France. With a brief pause in spread, this virus has again become active in countries like China, Japan, Australia and South Korea. Saudi Arabia recently cancelled flights to 15 countries in order to prevent spread of virus in the country. Thus, the statistics are increasing day-by-day^2^. The pandemic, which has affected the whole world, has no strange symptoms but rather common ones like tiredness, dry cough, fever and difficulty in breathing. Some other symptoms have also been experienced by a few infected persons like swelling in toes, running nose, sore throat, diarrhoea, ache and pain and nasal congestion^3^. The first wave showed severe effect on population above 50 whereas the second wave majorly impacted middle age groups. The third wave was comparatively mild, but spread massively. The ever-mutating nature of virus has left researchers baffled. Further, it has been reported that patients with hypertension and diabetes mellitus are at increased risk for CoViD19^4^.

In India and many other countries, reports have shown that patients have recovered from CoViD19 with the help of plasma therapy^5,6^. During this therapy, patients are treated using plasma donated by those patients who have already recovered from this virus. Some vaccines have also been produced by various countries. In India, Covaxin, Covishield and Sputnik V are being used to vaccinate its population^7^. USA is using vaccines like Pfizer, Moderna and Johnson & Johnson out of which Johnson & Johnson was previously banned for 11 days as recipients suffered from a dangerous blood clot^8^. Still a few countries are under lockdown across the world. A nasal spray called SaNOtize founded in 2017^9^ has won an approval as an option for early treatment of CoViD19 in February, 2022. India was praised by WHO, for taking the decision of lockdown, as “tough and timely”^10^. But, in the second wave, India also faced serious problems with almost four lacs cases being reported each day during the peak. Despite a successful and massive vaccination drive in the year 2021, the latter part of the year showed rising cases of virus owing to Omicron variant of CoViD19. 43.14 million cases have been observed in India till date, with 524,507 number of deaths. Due to the continued spread of virus, researchers from around the globe have realized the importance of carrying out active research in the field and have relentlessly focused their attention towards it^11–13^. Everyone understands that the need of the hour is to come up with solutions to contain and control this globe-spread virus.

The remaining paper is organized in the following manner: Section II gives a brief idea about the literature survey of this pandemic as well as research carried out to tackle the problem. Section III gives the details about the role of machine learning in classification of Chest X-ray images and briefly describes different models. Section IV details the proposed technique explaining feature extraction method with various classifiers and the database utilized to prove the efficacy of machine learning in CoViD19 with various tested methods. Section V discusses the experimental results followed by comparison of proposed method with other existing state-of-the-art methods in Section VI. At last, Section VII concludes the paper and discusses briefly about the future work that can be possible in machine learning to cater situations like this pandemic.

## Related work

In the past two years, a lot of research has been conducted on this novel virus. In some studies, it has been stated that CoViD19 is associated with pneumonia and can be cured using a drug related to malaria. This drug, called as chloroquine or hydroxychloroquine has been recommended by National Health Commission of the People’s Republic of China. But, it is able to cure patients who are at low risk^14^. As a side-effect of CoViD19, a fungal infection called as mucormycosis or black fungus has also been observed in a few patients in India post CoViD19 recovery^15,16^. Since the virus spreads through contact, an obvious solution to this problem has seemed to be the development of learned machines that can minimize human contact and ease out diagnostic procedures for doctors. The novel virus impacts lungs and causes heaviness in breathing. So, CT scans are being used by medical community to monitor the condition of patients from time to time. Automatic detection and classification of CT scans^17^ can lift off the burden from the diagnosticians and speed up the process of identifying potential patients. Apostolopoulous and Mpesiana^18^ have evaluated the performance of CNN architectures, which are a popular choice for medical image classification, pertaining to automatic detection of Coronavirus disease through X-rays^19^. The dataset for their research included 1427 X-ray images including normal conditions, confirmed Corona cases as well as common bacterial pneumonia cases. They have applied Transfer Learning strategy and CNN for classification. Based on the results, it has been suggested that X-ray imaging with deep-learning can be used for diagnosis of Coronavirus. Singh and Bansal^20^ have proposed the use of a deep learning model namely truncated Visual Geometry Group from Oxford (truncated VVG16) for screening of CT scans. After extraction of features, they have implemented principal component analysis (PCA) for feature selection. Once the useful features are available, they have used four learning models namely extreme learning machine (ELM), online sequential ELM, deep CNN and bagging ensemble along with SVM as classification models. On a dataset of 208 images, the last classifier has achieved an accuracy of 95.7% and performs better than other classifiers. Use of multi-objective differential evolution (MODE) and CNN has been advocated by Singh et al*.* in^21^. Instead of using random valued parameters for CNN, the researchers have proposed fine tuning of the initial parameters with the help of MODE. They have compared the results with CNN, adaptive neuro-fuzzy inference systems, and artificial neural networks and shown that their technique performs with a good accuracy rate. Sethy and Behera^22^ have also suggested deep learning based methodology for classification of X-ray images as Corona positive or negative. They have obtained validated data images from Kaggle, Github and Open-i. The Resnet 50 plus SVM model proposed by them achieves accuracy of 95.38%. A deep CNN model DeTraC (decompose, transfer and compose) has been validated and adapted by Abbas et al*.*^23^ for classification of chest X-rays for identifying COVID19 positive cases. They have also discussed the importance of transfer learning in situations where availability of annotated medical images in limited. Their deep CNN model achieves accuracy, sensitivity and specificity of 95.12%, 97.91% and 91.87% respectively. Detection of this infected virus based on the comprehensive dataset of CT scan and X-ray images collected from different sources that needs deep learning and transfer learning algorithms^24^. Wu et al*.* has developed novel idea to diagnose CoViD19 with the help of joint classification and segmentation (JCS) system in which they were able to detect CoViD19 chest CT scan. To develop the JCS system, they have built a high scale CoViD19 classification and segmentation dataset, with 350 uninfected cases and 144,167 chest CT images of 400 CoViD19 patients. Fine-grained pixel-level marks of opacifications, which are enhanced attenuation of the lung parenchyma, are annotated on 3,855 chest CT images of 200 patients^25^. Ahuja et al*.* detected CoViD19 using transfer learning from CT scan images decomposed to three levels using stationary wavelet in the proposed study. To improve detection accuracy, a three-phase detection model is proposed, with the following procedures: Phase 1: data augmentation using stationary wavelets, Phase 2: COVID-19 detection using a pre-trained CNN model, and Phase 3: abnormality localization in CT scan images. For the experimental evaluation, this work used well-known pre-trained architectures such as ResNet18, ResNet50, ResNet101, and SqueezeNet^26^. Umar et al*.* also used the transfer learning method for the virus detection in lungs. Authors have utilized the pre-trained Alexnet model for the proposed method and achieved satisfactory results^27^. Zhu et al*.* introduced a joint classification and regression approach to assess if the patients will experience CoViD19 symptoms in the future or not^28^. Hussain et al*.* proposed a technique named as Corona Detection (CoroDet) in which 2-, 3- and 4-class classifications were performed, where author claimed 99.1%, 94.2% and 91.2% of accuracy respectively^29^. Some more deep learning models are also introduced by the researchers which also includes pre-trained networks of deep learning^28,30–32^.

### Motivation

In the past year, various transfer learning models have been implemented for CoViD19 prediction^33^. These include ResNet 32 based model^34^, VGG19 model^35^, ResNet50 based model^36^, AlexNet based and Bidirectional Long Short-Term Memories (BiLSTM) layer based model^37^, DenseNet201 based model^38^, Deep-COVID (ResNet18, SqueezeNet and DenseNet121)^26,39^,five deep convolutional neural networks (AlexNet, VGGNet16, VGGNet19, GoogleNet, and ResNet50)^40^. All these studies have utilized the pre-trained convolutional neural network models for classification of CoViD19 chest X-ray images. All these transfer learning models achieved very good results in terms of accuracy. But, while taking the computational time into consideration, these models take a large amount of time.

### Contribution

At the moment, even after so many efforts, the virus is not completely contained. So, there is a need to detect CoViD19 virus in patients with great accuracy and in lesser time. Deep learning techniques are efficient but take more time in execution because of their iterative nature. Currently, with new cases being reported in some countries, leading to lockdown in some cities, there must be a faster solution for the prediction of CoViD19+ve patients. To deal with the situation at hand, some methods need to be developed which can help in detecting this virus without coming in contact with the patient with higher accuracy and lesser time. This is possible by using non-iterative approach in detecting the CoViD19.

## Machine learning in CoViD19

Machine learning helps in making a system, which is trained with the help of some data, detect and identify the patterns and make decisions with minimum level of human intervention^41,42^. As an effective vaccine has not yet been invented for the virus, it is a must to detect this virus in patients at an early stage. Since the virus is transmitted by touching and breathing close to another individual, it is imperative that a person should not come in contact with the patients. Under given circumstances, machine learning is one of the best solutions for the detection of CoViD19. Preliminaries used during this experimentation have been briefly discussed below.

### Support vector machine

**Figure 1:**
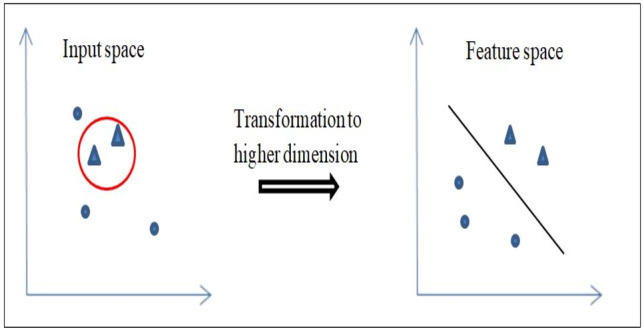
Basic idea of SVM.

Any non-linear problem can be converted into linearly separable problem in a higher dimensional space. This is the key point on which Support vector machines work. It maps the given input training data to a higher dimension feature space and then finds a hyperplane which maximizes the margin between two classes as shown in Fig. 1. As a result, the decision boundary in the input space is of non-linear nature. By using kernels, the separating hyperplane can be computed without explicitly transforming the input space to a higher dimensional feature space^43^.

What makes SVM a favourable choice is that it works well even when the training data available is small in size. Further, they are tolerant to imbalance in the number of training samples of two classes, which usually is the case^44^.

### Convolutional neural network

**Figure 2:**
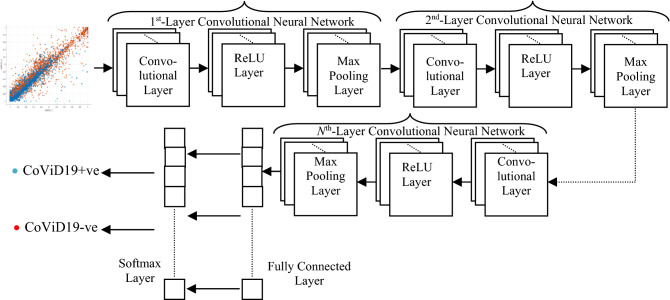
N-layer convolutional neural network architecture.

Problem with simple feedforward neural network is the use of huge number of neurons for their operation which results in large amount of execution time requirement. This problem is resolved with the help of convolutional neural network (CNN). It is also a type of deep neural network but what it performs is that it extracts the image features by reducing the dimensions of the image without even losing their characteristics^45^. A single layer CNN consists of three main sub-layers i.e. convolutional layer, ReLU layer and max pooling layer. Architecture for Nth-layer CNN is shown in Fig. 2.

#### Convolutional layer

In this layer of CNN, features are extracted from the image by convolving a filter with the part of image (having size same as that of filter). This process is repeated over whole image by sliding the filter using a Stride and features are obtained.

#### ReLU layer

ReLU is rectifying linear unit. It is an activation function that makes all negative values of the resultant obtained from convolutional layer to zero. Its function is shown below:

1 $$h\left(y\right)=\mathrm{max}(0,y)$$

#### Max pooling layer

**Figure 3:**
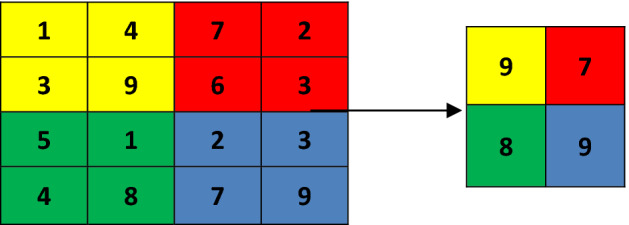
Max pooling layer with f = 2 × 2 and s = 2.

Max pooling is the most common non-linear down sampling process of CNN. It is a necessary step before applying next convolutional layer in CNN as it reduces the dimensions of the image and hence, computational cost can be reduced. In this layer also, a filter ‘f’ is chosen which is slide over the image with Stride ‘s’. Filter size image field is selected and maximum value of that portion is obtained by non-overlapping sliding of the filter over the image. It is shown in an example in Fig. 3.

Therefore, CNN is also helpful in dimension reduction as well, keeping the important information within the resultant image. It is utilized here for binary classification of chest X-ray images.

### Kernel extreme learning machine

Extreme learning machines have gained attention widely because of their simplicity. They have a single hidden layer, which does not need to be tuned. Since their proposal in 2006^46^, they have been extended to adopt a more generalized form, where the neurons in a single layer feed-forward network (SLFFN) need not be alike^47–49^.

For a SLFFN, let there be a dataset of H samples {*s*_*i*_, *o*_*i*_} i = 1,2,…,*M*, where *s*_*i*_ is the ith training input vector of dimension *D* and *o*_*i*_ is the corresponding class label belonging to either one of the classes {1, 2, …, *L*}. Hence the dimension of input space for SLFFN is *D* and the dimension of output layer is *L*. The input space is first mapped to a higher dimensional space that has the dimension equal to number of neurons in the hidden layer (here, say ${\ddot{\text{H}}}$). The important features are preserved during this mapping. Next phase is the projection of high-dimension feature space to a low-dimension feature space so as to map the output from H neurons to L output classes^50^.

Thus, the system can be modelled using Eq. (2) as given below:

2 $$\mathop \sum \limits_{i = 1}^{\ddot{\text{H}}} {\mathcal{B}}_{i} A_{i} \left( {s_{j} } \right) = \mathop \sum \limits_{i = 1}^{\ddot{\text{H}}} {\mathcal{B}}_{i} A\left( {\varpi_{i} .s_{j} + b_{i} } \right) = e_{j}$$

where A(s) is the activation function of the hidden layer, ${\mathrm{\varpi }}_{\mathrm{i}}={[{\mathrm{\varpi }}_{\mathrm{i}1}, {\mathrm{\varpi }}_{\mathrm{i}2}, \dots , {\mathrm{\varpi }}_{\mathrm{iD}}]}^{\mathrm{T}}$ is the weight vector connecting the inputs to the ith hidden neuron, ${\mathcal{B}}_{\mathrm{i}}={[{\mathcal{B}}_{\mathrm{i}1}, {\mathcal{B}}_{\mathrm{i}2}, \dots , {\mathcal{B}}_{\mathrm{iL}}]}^{\mathrm{T}}$ is the weights connecting hidden layer neurons to output layer nodes, and bi is the bias. The output layer is assumed to have a linear activation function.

According to Huang et al.^46^﻿, a SLFFN with Ḧ nodes in the hidden layer and an activation function *A*(*s*) can achieve zero error for *H* sample by approximation such that,

3 $$\mathop \sum \limits_{j = 1}^{\ddot{\text{H}}} ||e_{i} - o_{j} || = 0$$

For *H* samples, Eq. (2) can thus be written as,

4 $${\varvec{M}}\mathcal{B}={\varvec{O}},$$

where,

$${\varvec{M}} = \left[ {\begin{array}{*{20}c} {A\left( {\varpi_{1} .s_{1} + b_{1} } \right)} & \cdots & {A\left( {\varpi_{{{\ddot{\text{H}}}}} .s_{1} + b_{{{\ddot{\text{H}}}}} } \right)} \\ \vdots & \ddots & \vdots \\ {A\left( {\varpi_{1} .s_{H} + b_{1} } \right)} & \cdots & {A\left( {\varpi_{{{\ddot{\text{H}}}}} .s_{H} + b_{{{\ddot{\text{H}}}}} } \right)} \\ \end{array} } \right],{\mathcal{B}} = \left[ {\begin{array}{*{20}c} {{\mathcal{B}}_{1}^{{\text{T}}} } \\ \vdots \\ {{\mathcal{B}}_{{{\ddot{\text{H}}}}}^{{\text{T}}} } \\ \end{array} } \right],{\varvec{O}} = \left[ {\begin{array}{*{20}c} {o_{1}^{{\text{T}}} } \\ \vdots \\ {o_{H}^{{\text{T}}} } \\ \end{array} } \right].$$

The minimum norm least square solution of SLFFN can be given by:

5 $${\mathcal{B}}^{\prime} = {\varvec{M}}^{\prime}{\varvec{O}}$$

where ***M***′ is the generalized inverse (Moore–Penrose) of ***M.***

Unlike other machine learning algorithms, what makes extreme learning machines stand out is their non-iterative behaviour. They are highly scalable and have comparatively less computational complexity^51^.

**Figure 4:**
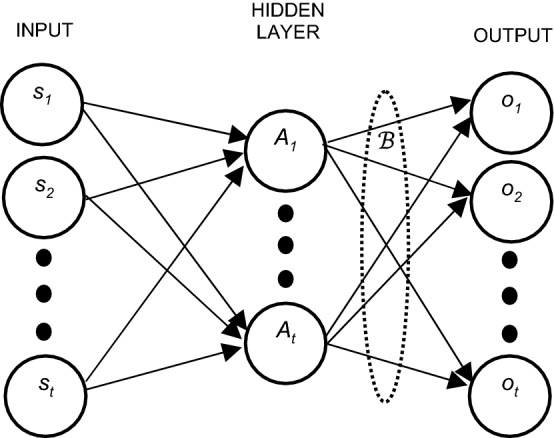
KELM architecture.

Kernel extreme learning machines are an extension of ELM. Here, the hidden layer outputs are calculated once and stored permanently in the kernel matrix. It is not calculated on the output layer of dimensionality L, but rather on the input data dimension and samples^52,53^. It is shown is Fig. 4.

KELM has the capability of approximation as well as classification. Single model can be thus used for variety of applications. In the propose scheme, KELM is used as a binary classifier as only CoViD19+ve and CoViD19−ve are the two classes which are classified using this classifier.

## Proposed technique

This section begins with a detailed discussion on the database used for checking the efficacy of proposed technique. Next, pre-processing as well as feature extraction is elaborated as it is an important step before formation of dataset for KELM. Also, the steps of implementing the proposed technique are also discussed in this section.

### Database used

**Figure 5:**
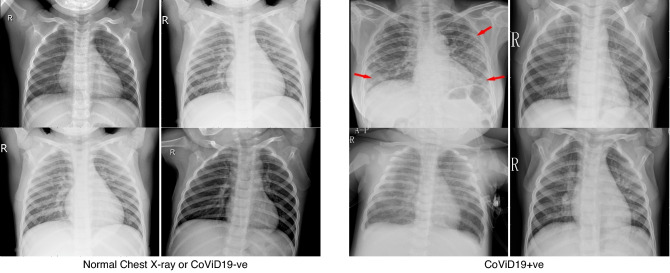
Chest X-ray of normal (CoViD19−ve) and CoViD19+ve.

**Figure 6:**
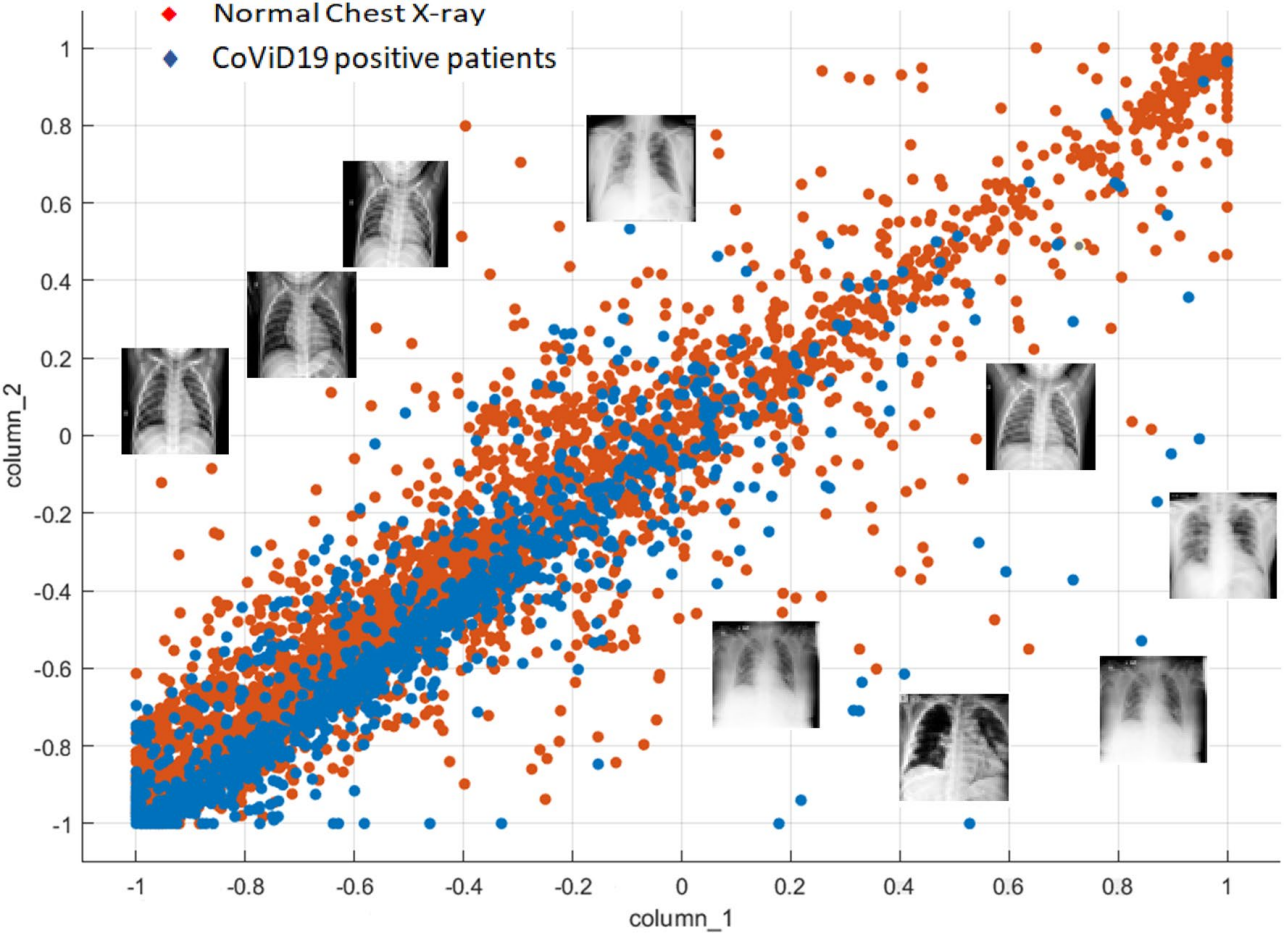
Scatter plot of the chest X-ray data showing normal (CoViD19−ve) and CoViD19+ve patients.

As CoViD19 is nothing but associated pneumonia, therefore, chest X-ray of an individual can be helpful in detecting whether the person is CoViD19 positive or not. The database consists of 5,856 images of chest X-ray having 4,273 CoViD19 positive images and 1,583 normal images. The X-ray images are gray scale in nature and have different dimensions. Hence, all the images have been resized to dimension 50 × 50. In terms of pixel values, images are normalized between − 1 and + 1. Sample images are shown in Fig. 5 in which the chest X-ray of the normal (CoViD19−ve) and CoViD19 associated pneumonia (CoViD19+ve) are shown. It is a publicly available database^54^. Normal chest X-ray is a clear image of lungs without any areas of opacification. CoViD19 associated pneumonia is detected by interstitial pattern as depicted with red arrows. But, this difference may seem insignificant to an untrained person. A scatter plot of all the images is also shown in Fig. 6 showing the distribution of the database. Red dots are representing the normal chest X-ray images and blue dots are denoting the CoViD19 associated pneumonia images of the patients. The x-axis and y-axis in Fig. 6 are particular pixel values of all the samples of the database. As all images (samples) have been resized to 50 × 50, hence, there are 2500 pixel values for each image in the database which are converted into a vector from its matrix representation. Thus, from all the images, a dataset is formed consisting of 5856 rows and 2500 columns. Figure 6 only represents correlation among column 1 (x-axis) and column 2 (y-axis) of all the samples. Some X-ray images have been added in Fig. 6 along with red and blue dots to showcase that the images are indistinguishable for untrained people and may seem similar to them.

**Figure 7:**
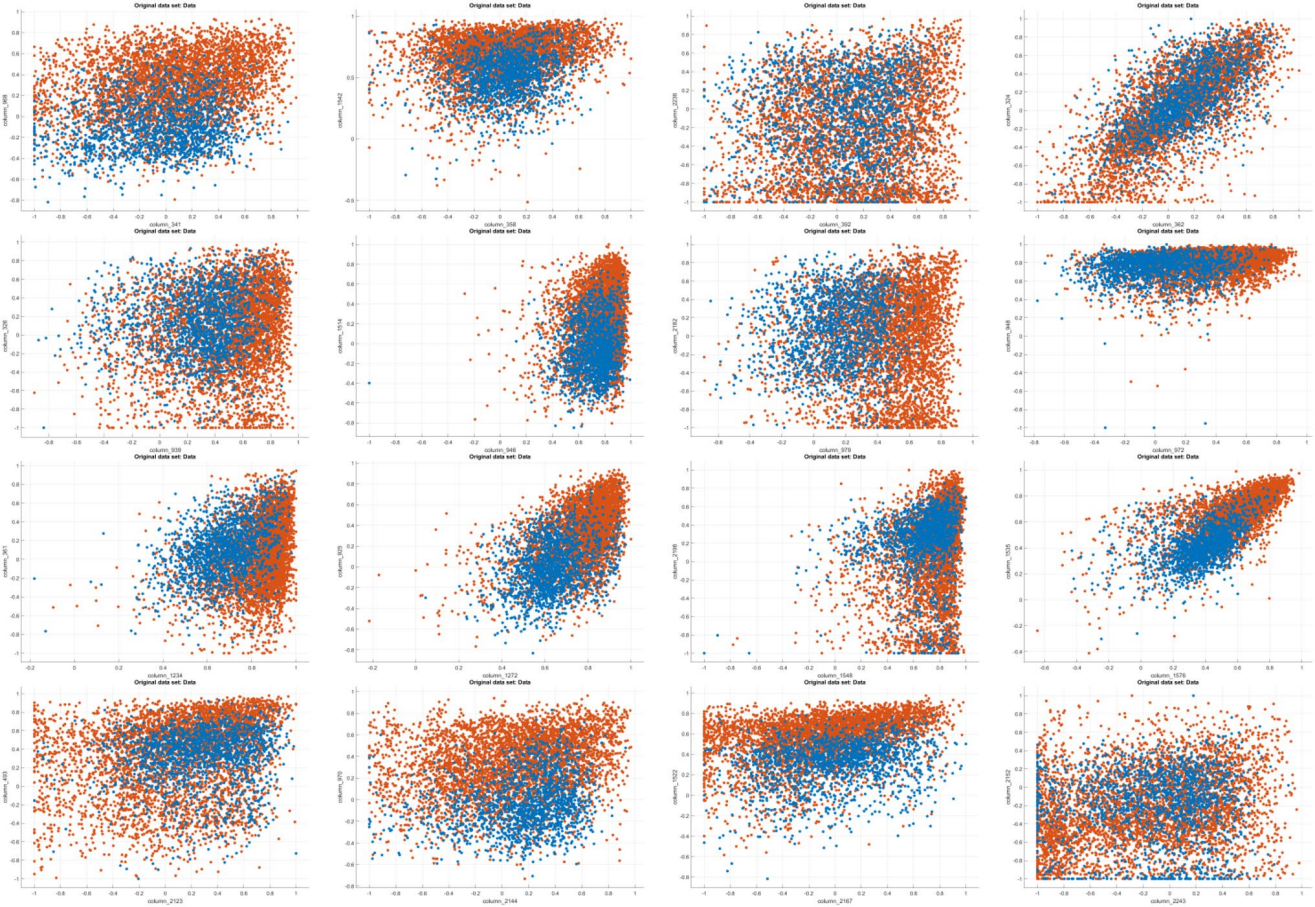
Scatter plots of chest X-ray data with different combinations of pixel value columns. ^1^The x and y-axis of scatter plots are the column numbers out of 2500 columns in the dataset namely column 341 and column 968, column 358 and column 1542, column 392 and column 2236, column 362 and column 324, column 939 and column 326, column 946 and column 1514, column 979 and column 2182, column 972 and column 948, column 1234 and column 361, column 1272 and column 925, column 1548 and column 2198, column 1576 and column 1535, column 2123 and column 493, column 2144 and column 970, column 2167 and column 1522, column 2243 and column 2152 respectively.

Similarly, some more scatter plots have been shown in Fig. 7. These scatter plots have been plotted by taking various random combinations of pixel values of all the images. The column numbers are written on x-axis and y-axis of respective images^1^. The scatter plots depict normalized pixel values between (− 1.0) to (+ 1.0). The basis of showing these scatter plots is only that X-ray images of normal people and CoViD19+ve patients are so similar that it is difficult for an untrained person to differentiate between the two.

This database is split sequentially into train and test data on the basis of train-test ratio of 10–90, 30–70, 50–50 and 70–30. Therefore, train data will have 158, 475, 791 and 1108 images from normal chest X-ray or CoViD19−ve images and 427, 1282, 2136 and 2991 images from CoViD19 positive patients (CoViD19+ve) respectively. This makes 585, 1757, 2215 and 4099 images (for 10–90, 30–70, 50–50 and 70–30) in total for train data. Therefore, remaining 5271, 4099, 2929 and 1757 images are utilized as test data (1425, 1108, 792 and 475 images of normal chest X-ray and 3846, 2991, 2137 and 1282 of CoViD19 positive patients) for 10–90, 30–70, 50–50 and 70–30 respectively.

### Preprocessing and feature extraction

**Figure 8:**
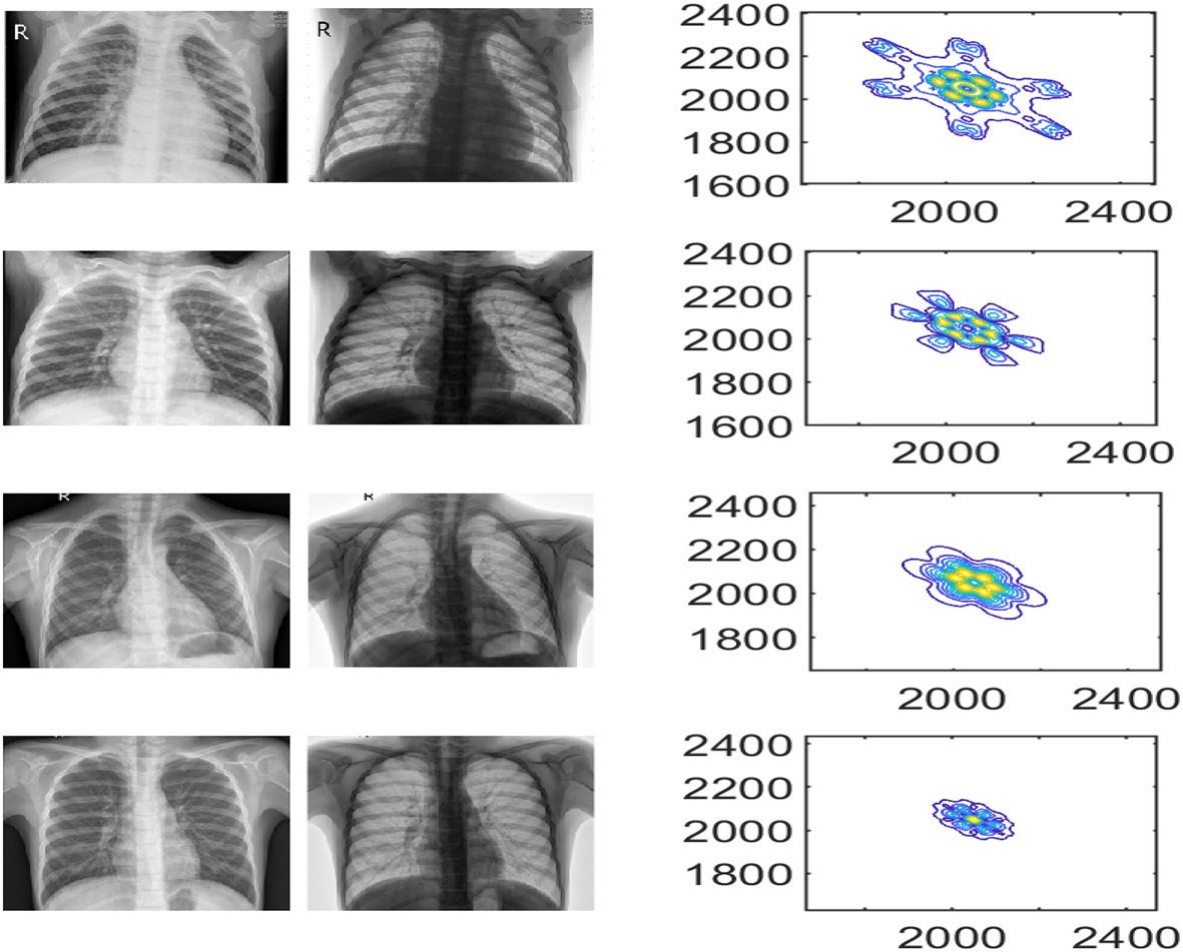
CoViD19+ve (first two rows) and CoViD19−ve (last two rows).

The images of chest X-ray are pre-processed by enhancing the image as shown in Fig. 8 (second column). Four sample images are represented (two samples of CoViD19+ve in first two rows) and (two images of CoViD19−ve in last two rows). The last column of the figure shows the extraction of features from images. This extraction of features, from chest X-ray enhanced images, is performed with the help of a higher order statistics parameter like Bispectrum.

Higher order statistics can be defined in terms of moments and cumulants. Cumulants are non-linear combinations of various moments. The motivation behind using higher order statistics is to analyze the disturbances in chest X-ray images of the patients due to the attack of CoViD19. Bispectrum is the Fourier transform of third order cumulant^55,56^.

The third-order cumulant ${K}_{3}\left({\epsilon }_{1},{\epsilon }_{2}\right)$ is represented as

6 $${K}_{3}\left({\epsilon }_{1},{\epsilon }_{2}\right)={n}_{3}\left({\epsilon }_{1},{\epsilon }_{2}\right)-{n}_{1}\left[{n}_{2}\left({\epsilon }_{1}\right)+{n}_{2}\left({\epsilon }_{2}\right)-{n}_{2}\left({\epsilon }_{1}-{\epsilon }_{2}\right)\right]+2{({n}_{1})}^{3}$$

In this, ${n}_{3}\left({\epsilon }_{1},{\epsilon }_{2}\right)$ depicts the third-order moment. ${K}_{3}\left({\epsilon }_{1},{\epsilon }_{2}\right)$ is the third-order cumulant that explains the skewness of the data and is equal to ${n}_{3}\left({\epsilon }_{1},{\epsilon }_{2}\right)$ for zero-mean. Skewness is a measure of asymmetry of any distribution about its mean. Positive value of skew indicates that the tail on the left side of the data, is shorter and thicker than the right side. In cases where one tail is short but the other tail is thick, skewness does not obey a simple rule. A zero value shows that the tails on both sides of the mean is balanced, which is the case for a symmetric distribution. It is also true for an asymmetric distribution where the asymmetries, such as one tail being short but thin, and the other being long but thick.

If these cumulants are considered in frequency domain, then it can be obtained by taking the Fourier transform of these cumulants. Fourier transform of third-order cumulant is given as:

7 $$S\left({\varphi }_{1},{\varphi }_{2}\right)=Z\left({\varphi }_{1}\right)Z\left({\varphi }_{2}\right){Z}^{*}\left({\varphi }_{1}+{\varphi }_{2}\right)=\sum_{{u}_{1}=-\infty }^{\infty }\sum_{{u}_{2}=-\infty }^{\infty }{K}_{3}\left({\epsilon }_{1},{\epsilon }_{2}\right).{e}^{-j\pi ({\varphi }_{1}{u}_{1}+{\varphi }_{2}{u}_{2})}$$

where $S\left({\varphi }_{1},{\varphi }_{2}\right)$ is the Bispectrum of z(m), ${K}_{3}\left({\epsilon }_{1},{\epsilon }_{2}\right)$ is the third-order cumulant and $Z\left(\varphi \right)$ is the Fourier transform of x(n).

We demonstrate that bispectral analysis has great potential in this new application where bicoherence magnitude responses of the image are used to identify chest X-ray images. Bispectrum is often used for detecting the existence of quadratic correlation within a signal, as being applied in oceanography, EEG signal analysis, manufacturing, non-destructive structural fatigue detection and plasma physics applications^57^. We compute the bispectrum magnitude response for the chest X-ray images for different class of images and observe that the distributions for the CoViD19+ve images are greater than that of CoViD19−ve images as shown in Fig. 8.

### Steps of implementation

**Figure 9:**
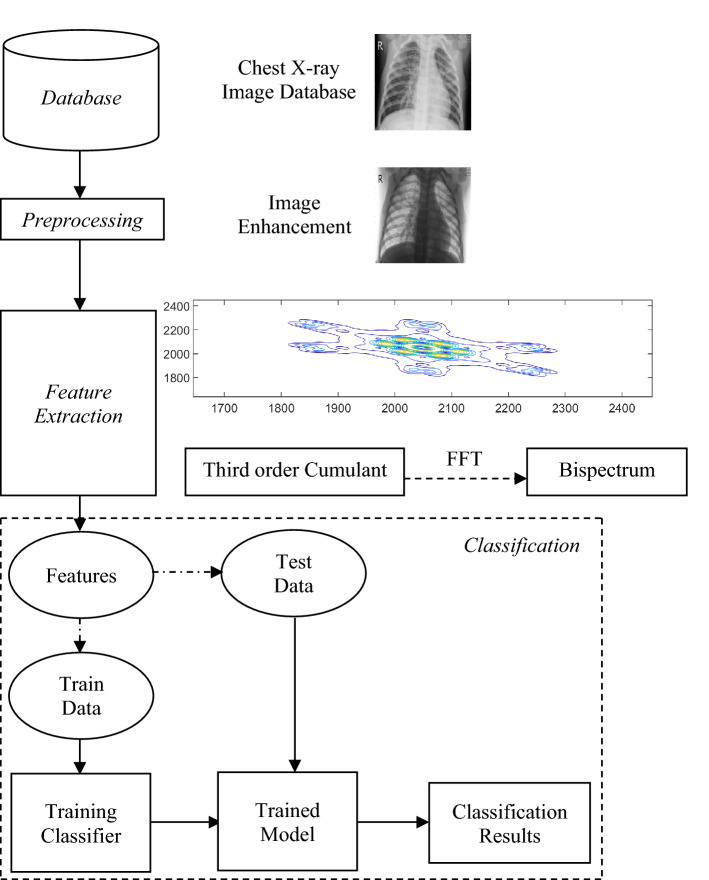
Block diagram of workflow in non-iterative approach of KELM.

A basic block diagram of workflow for non-iterative approach using KELM is shown in Fig. 9. The steps for implementing proposed technique are as follows:

1. Select CoViD19 chest X-ray images and resize them to size 50 × 50. The images in dataset are of variable size, and hence need to be made coherent to a uniform size and dimension.
2. Enhance the images using adaptive histogram equalization^58^. Pre-processing techniques lead to data enhancement and refinement by identifying the affected part in the chest X-ray images^59^.
3. Perform feature extraction using third-order cumulants.
4. Apply fast Fourier transform on third-order cumulants to obtain bispectrum of the images. It helps in taking out features that includes color, texture information in the images^60^.
5. Normalize data between values − 1.0 and + 1.0.
6. Divide these normalized features into train and test data. Train data helps in training of the classifier and thereafter, a trained model is obtained. There are multiple available classifiers in machine learning like kNN^61^, SVM^62,63^, artificial neural network (ANN)^64^, etc. We have implemented SVM, CNN and KELM.
7. Provide test data features to the trained model for testing and hence, derive the classification results.

## Experimental results and discussion

For all the experiments, the images of the Chest X-ray database have been resized to 50 × 50 and converted to gray scale. All the images gone through the image enhancement process and bispectrum features are obtained from all the enhanced images. The feature vector set size remains same as the size of the resized image (50 × 50 matrix – 1 × 2500 vector). The features computed from the enhanced chest X-ray image database is experimented over three classifiers viz. SVM, Convolutional Neural Network and kernel extreme learning machine (KELM), which is a non-iterative learning machine model. SVM and Convolutional Neural Network are the iterative learning approaches which are utilized here to compare the execution time of the iterative learning approaches with non-iterative learning approach. The performance of these classifiers is measured mainly on three parameters based on confusion matrix. They are briefly explained here.

Confusion matrix is defined as the matrix using which correctness of a classifier can be measured. It is represented below and its parameters are defined as:

**Table:** 

*tp*	*fn*
*fp*	*tn*

*tn* (true negatives)—number of correctly identified instances that do not belong to the class.

*tp* (true positives)—number of correctly identified instances that belong to the class.

*fp* (false positives)—number of instances that were incorrectly assigned to the class.

*fn* (false negatives)—number of instances that not identified as a class instance.

As we know the output is either CoViD (+ ve) or Normal (−ve). The three parameters for identifying the output are briefly explained below:.

- Accuracy: It is defined as ratio of the total number of accurate predictions and total number of predictions.
  8 $$\mathrm{Accuracy}=\frac{tp+tn}{tp+tn+fp+fn}$$
- Sensitivity: It is also known as recall and defined as the ratio of correctly identified (*tp*) cases and summation of *tp* & *fn*.
  9 $$\mathrm{Sensitivity}=\frac{tp}{tp+fn}$$
- Specificity: It is the ratio of number of correctly identified cases that do not belong to the class (*tn*) and the summation of *tn* & *fp*.
  10 $$\mathrm{Specificity}=\frac{tn}{tn+fp}$$

Results computed on the fore-mentioned classifiers based on these performance metrics are here as follows.

## Results with SVM classifier

SVM is utilized with train-test ratio of 10–90, 30–70, 50–50 and 70–30 as shown in Table 1. Quite good percentage accuracy of 95.85% is achieved on 70–30 train-test ratio. SVM gives such good results with quadratic kernel, penalty parameter taken as 1, 0.001 of tolerance, and value of degree as 3 during experimentation. SVM performs very well but have very high execution time because of iterative learning approach. It can be seen in Table 1.

**Table 1:** Performance measures obtained on SVM classifier.

Performance measures	Train-test ratio			
	10–90	30–70	50–50	70–30
Accuracy (%)	90.32	94.36	95.29	95.85
Sensitivity (%)	91.02	96.03	92.24	96.00
Specificity (%)	90.07	93.75	96.38	95.79
Execution time (s)	22.491	162.89	377.87	422.4

## Results with CNN classifier

Table 2 shows the performance of 2-Layer CNN on the bispectrum features of CoViD19 chest X-ray images. In both the layers of CNN, 3 × 3 size of filter is utilized in convolutional layer and 2 × 2 size of mask in max pooling layer. The results achieved are excellent in terms of accuracy as 96.81% accuracy is achieved with 70–30 train-test ratio. The execution time for this set is 215 s which is better than the previous classifiers used in the experimentation.

**Table 2:** Performance measures obtained on 2-layer CNN classifier.

Performance measures	Train-test ratio			
	10–90	30–70	50–50	70–30
Accuracy (%)	86.76	94.00	94.16	96.81
Sensitivity (%)	87.09	95.94	84.96	93.47
Specificity (%)	86.64	93.28	97.57	98.05
Execution time (s)	26	112	156	215

Table 3 is representing the results for 3-Layer CNN. Results achieved with this classifier are almost same as that achieved using 2-Layer CNN. A small disadvantage of using this classifier is increase in execution time which is due to the increase in one layer of CNN. Specifications of filter and stride are same as that used in 2-Layer CNN. Maximum percentage accuracy achieved with 3-layer CNN is 95.90% with 363 s of execution time.

**Table 3:** Performance measures obtained on 3-layer CNN classifier.

Performance measures	Train-test ratio			
	10–90	30–70	50–50	70–30
Accuracy (%)	86.93	94.46	94.43	95.90
Sensitivity (%)	75.79	94.13	86.22	89.89
Specificity (%)	91.06	94.58	97.47	98.13
Execution time (s)	43	151	285	363

All these classifiers utilized are iterative and hence, execution time is quite large. In CNN, only two and three layers are used and execution time is comparatively large. If transfer learning approaches are utilized, in which there are so many layers, results in very large execution time.

## Results with KELM classifier

KELM is a faster classifier that is non-iterative in nature. There are various kernel functions which can be used in the kernel-based ELM. They are linear, polynomial kernel, sigmoid kernel, wavelet kernel, and RBF kernel^65^. Any of these kernel functions can be utilized with KELM depending upon the requirement and hence, the kernel-based ELM model is defined as the kernel extreme learning machine.

Table 4 represents the variation in accuracy observed with various kernel functions. For this, regularization coefficient and kernel parameters are computed as 1 and 345 respectively. These parameters’ values were obtained experimentally by optimizing the accuracy using optimization algorithm. The table is shown below:

**Table 4:** Performance measures with different kernel functions obtained on KELM classifier.

Kernel function	Train-test ratio			
	10–90	30–70	50–50	70–30
RBF kernel	90.86	95.32	96.52	96.64
Linear kernel	82.05	85.56	89.48	91.12
Polynomial kernel	88.24	92.27	93.41	93.85
Wavelet kernel	49.57	49.52	50.22	48.72
Sigmoid kernel	86.67	91.19	82.35	83.72

It can be seen from the table that best accuracy is achieved with RBF kernel function and hence, RBF kernel function is utilized here for the results and comparing them with other state-of-the-art methods.

Table 5 shows the classification results for KELM classifier with RBF kernel function on CoViD19 database.

**Table 5:** Performance measures obtained on KELM classifier.

Performance measures	Train-test ratio			
	10–90	30–70	50–50	70–30
Accuracy (%)	**90.86**	**95.32**	**96.52**	**96.64**
Sensitivity (%)	82.86	88.81	93.37	91.60
Specificity (%)	93.85	97.94	96	98.65
Execution time (s)	**0.3166**	**0.9042**	**1.3034**	**1.5991**

Significant values are in bold.

For KELM also, train-test ratio is selected as 10–90, 30–70, 50–50 and 70–30 and an accuracy of 90.86%, 95.32%, 96.52% and 96.64% has been obtained respectively. It can be seen through the execution time (Table 5) that non-iterative nature of KELM gives an advantage of much faster speed.

## Comparison and discussion

**Figure 10:**
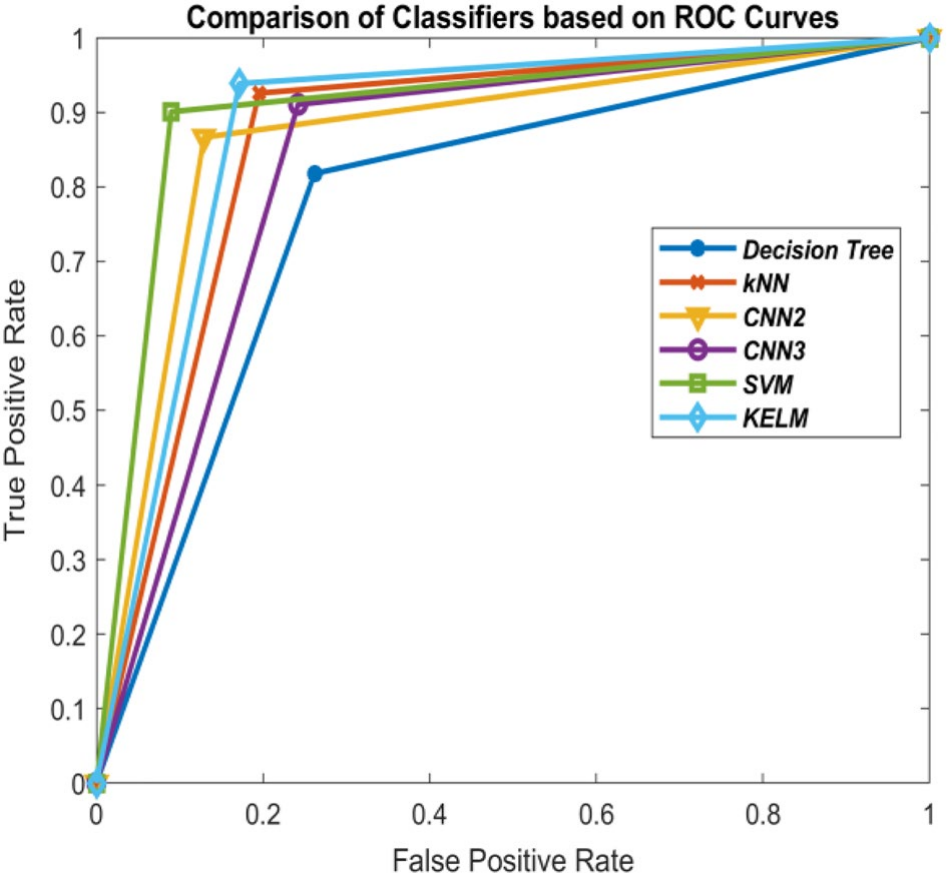
Comparison of the classifiers based on ROC curves (10–90 train-test ratio) of CoViD19+ve and CoViD19−ve.

**Figure 11:**
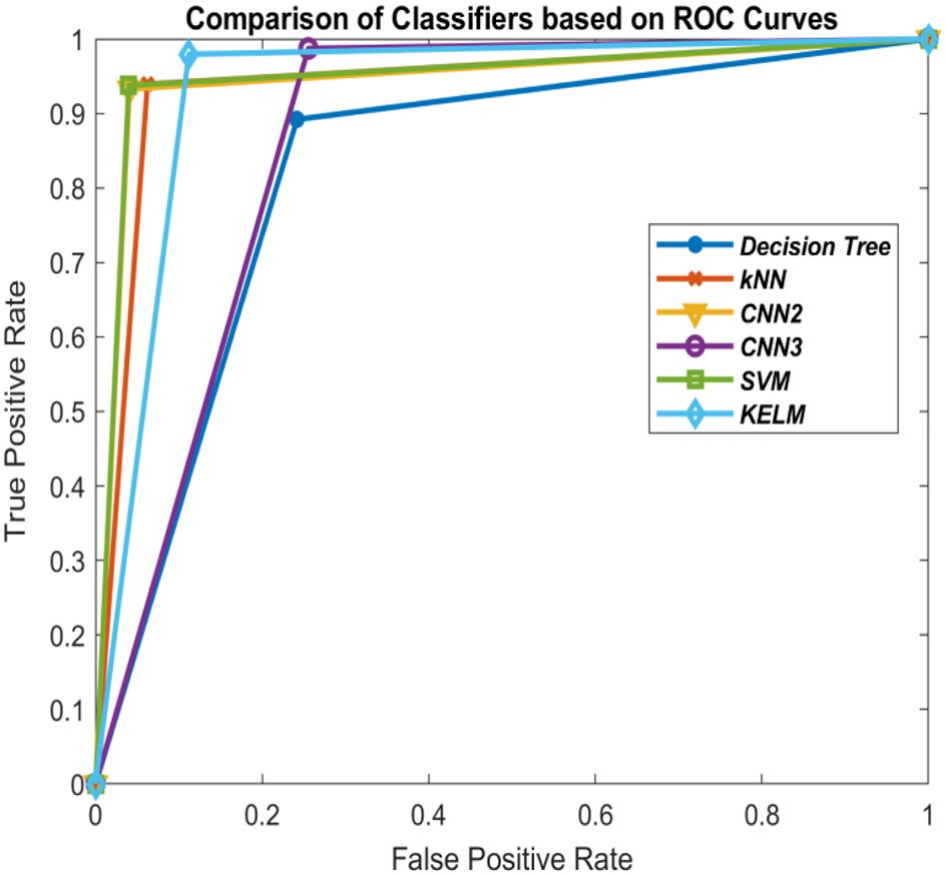
Comparison of the classifiers based on ROC curves (30–70 train-test ratio) of CoViD19+ve and CoViD19−ve.

**Figure 12:**
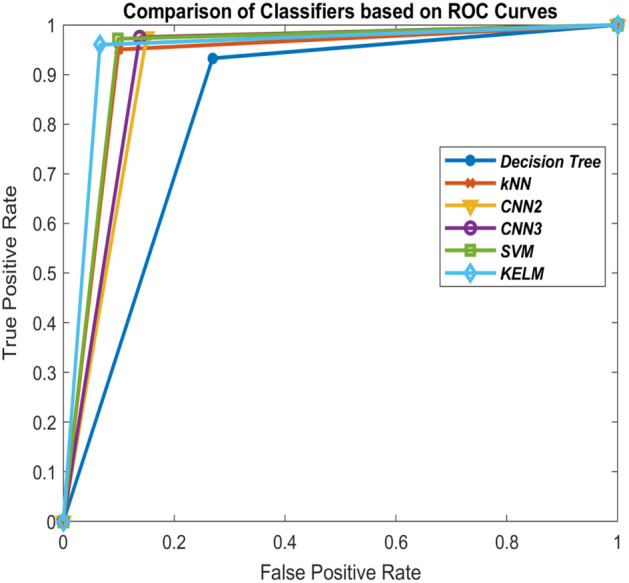
Comparison of the classifiers based on ROC curves (50–50 train-test ratio) of CoViD19+ve and CoViD19−ve.

**Figure 13:**
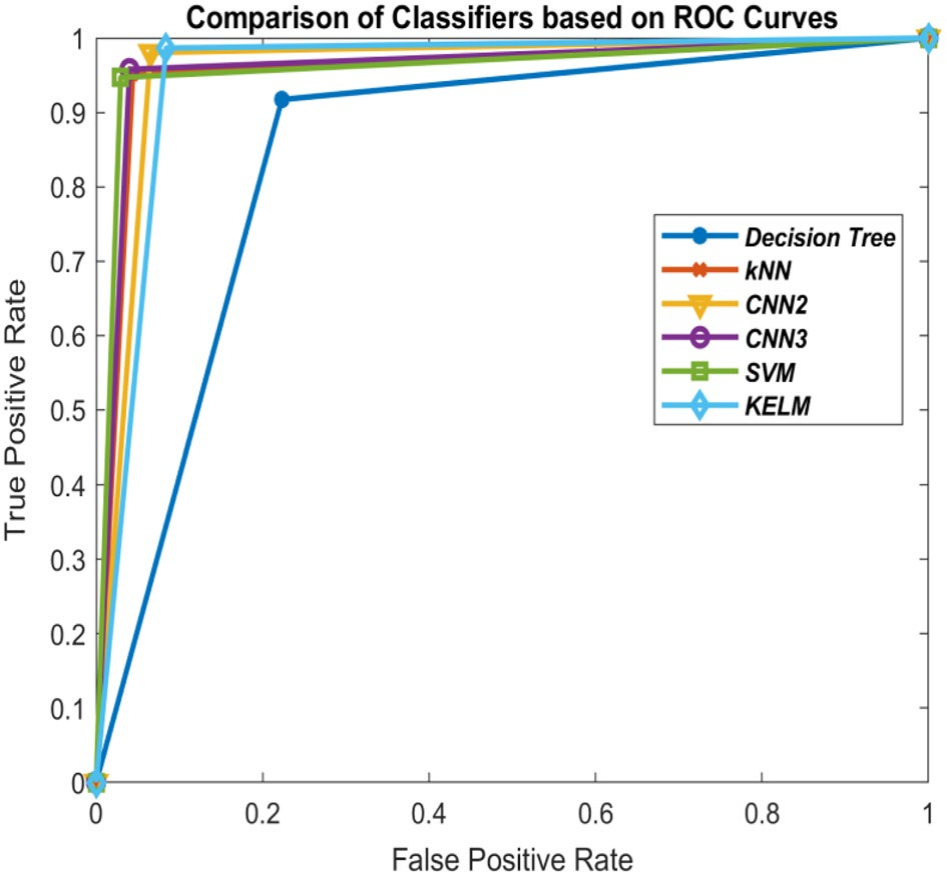
Comparison of the classifiers based on ROC curves (70–30 train-test ratio) of CoViD19+ve and CoViD19−ve.

All the classifiers with bispectral magnitude analysis of the chest X-ray images of CoViD19 patients utilized during experimentation provide very good results and it has already been seen in the previous sections. Now, in this section, results obtained from these classifiers are compared with each other and with the existing state-of-art methods on the chest X-ray database of CoViD19 (Table 6). Comparison of these classifiers is shown in Figs. 10, 11, 12 and 13 on the basis of their ROC curves.

**Table 6:** Comparison of the classifiers with other state-of-art approaches based on accuracy (%).

Method	Train-test ratio			
	10–90	30–70	50–50	70–30
KELM	***90.86***	***95.32***	***96.52***	***96.64***
3-layer CNN	86.93	94.46	94.43	95.90
2-layer CNN	86.76	94.00	94.16	***96.81***
SVM	90.32	94.36	95.29	95.85
kNN	89.30	93.90	93.68	95.28
Decision tree	79.62	85.58	87.81	87.93
ANN^21^	89.5	89.9	89.8	90.7
Deep transfer learning^34^	–	–	–	93.02
ANFIS^21^	91.4	91.5	91.2	91.9
DeTraC deep CNN^23^	–	–	–	95.12
Differential evolution based CNN^21^	92.2	92.45	92.7	93.2
Modified CNN using AlexNet^24^	–	–	94.1	–
CNN using ResNet50^66^	–	–	–	98.00 (95% train data)

Significant values are in bolditalic.

**Figure 14:**
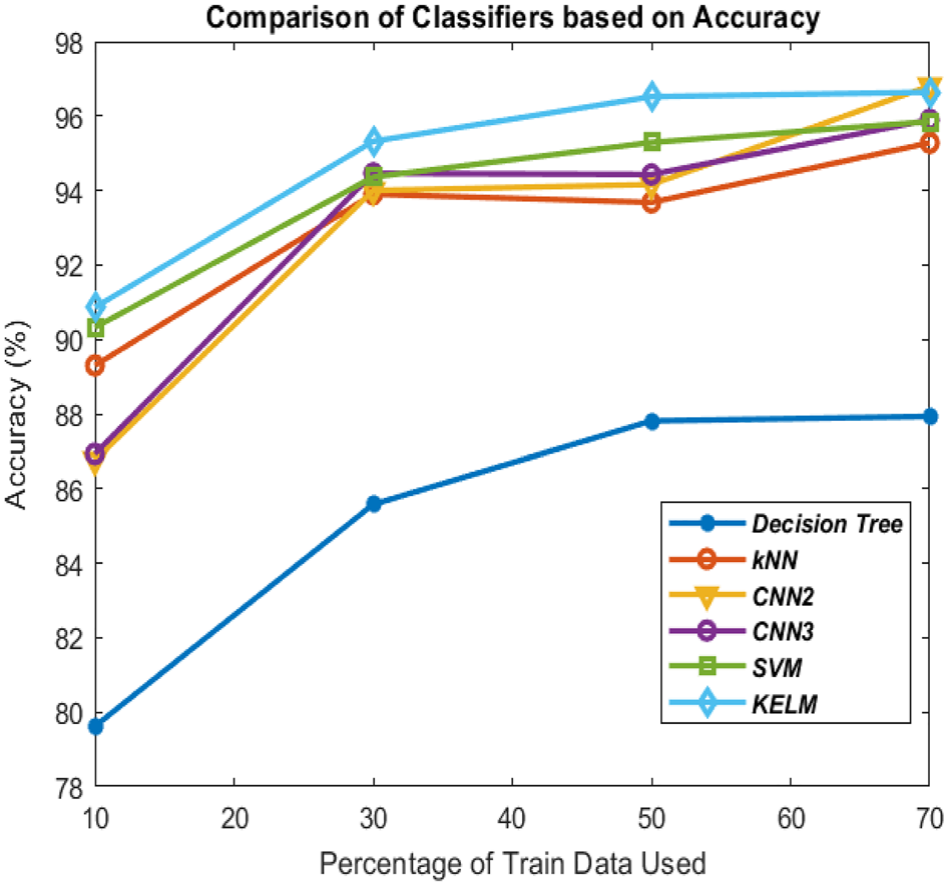
Comparison of the classifiers based on accuracy of recognizing CoViD19+ve and CoViD19−ve.

Tables 1, 2, 3 and 5 also show the values of performance measures, sensitivity and specificity. Sensitivity tells about the images of chest X-ray which are not affected with corona virus signifying CoViD19−ve images while specificity represents the chest X-ray images having the presence of corona virus. It can be seen from the tables that KELM performs best among all these classifiers in identifying CoViD19+ve and CoViD19−ve cases based on chest X-ray images. The same is also clearly visible in Figs. 10, 11, 12 and 13 which are plotted using the values of sensitivity and specificity of these classifiers. These figures are plotted for 10–90, 30–70, 50–50 and 70–30 train-test ratio respectively. Figure 14 represents the plots of classifiers used during experimentation based on their accuracies in classifying CoViD19+ve and CoViD19−ve images. It can be clearly seen that KELM performs best and Decision tree performs worst among these classifiers. 2-Layer CNN also performs equally as KELM but only when the train data is large.

**Figure 15:**
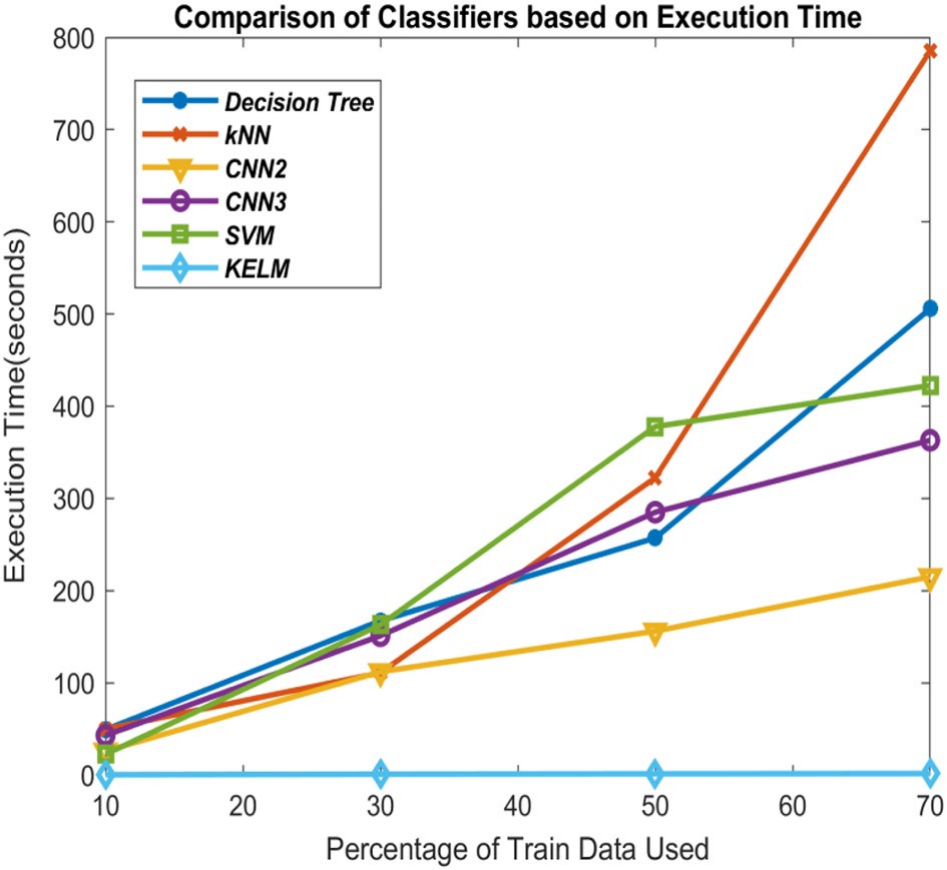
Comparison of the classifiers based on execution time of recognizing CoViD19+ve and CoViD19−ve.

As CNN is an iterative algorithm, therefore, increasing the training data results in the increase in their execution time as well. Similar case can be observed with transfer learning approaches used in the existing approaches where there are so many layers compared to 2-Layer CNN and 3-Layer CNN. KELM is non-iterative, hence, performs faster. It can be observed from Fig. 15. KELM is fastest among all the classifiers utilized during the experimentation followed by 2-Layer CNN and *k*NN is slowest. The results of these classifiers are compared with other state-of-art approaches and are shown in Table 6. In^21^ and^34^, classification was performed on Corona virus database based on differential evolution based CNN and deep transfer learning respectively. Both these papers have utilized comparatively lesser number of images in their experimentation. Narin et al.^66^ have achieved 98% accuracy in detecting the patients correctly but they have trained the model with 95% of the total data which might result in overtraining of the model leading to good results. One advantage that can be observed in our models is that they maintain high accuracy even with large number of test images, especially for KELM with very less computational complexity.

On the contrary, CNN based state-of-art approaches like ResNet, Inception, etc. also produce comparable results to KELM. A comparison table in which accuracy of KELM is compared with ResNet and Inception CNNs is shown in the Table 7. KELM performs classification with high accuracy even if less amount of data is used to train the classifier while CNNs require large amount of training data for good classification results as seen from Tables 2, 3 and 5. Also, all these CNN based approaches are iterative in nature and take large amount of time compared to KELM. Hence, KELM being a non-iterative algorithm comes out as very helpful, fast and efficient algorithm in this pandemic (CoViD19).

**Table 7:** Comparison of KELM with ResNet and other CNN based state-of-art approaches based on accuracy (%).

Method	Train-test ratio	Accuracy (%)
KELM	70–30	96.64
KELM	80–20	97.01
ResNet^67^	80–20	86.00
ResNet 50^68^	80–20	93.62
COVNet^69^	80–20	96.00
Inception v3^68^	80–20	93.62
Bayesian CNN^70^	80–20	92.90
Inception-ResNet v2^68^	80–20	88.59

## Conclusion and future scope

The results obtained in this experiment depicts that machine learning can be very helpful in dealing with this worldwide pandemic. Higher order statistics has provided a clear view in differentiating the CoViD+ve patients from the normal ones. As various classifiers are applied, they give very good results on the chest X-ray images of CoViD19 database. 2-Layer CNN performs the best with 96.81% of accuracy in classifying the CoViD19−ve chest X-ray images from CoViD19+ve images. But the execution time for 2-Layer CNN for this accuracy is 215 s. And when speed is the concern, as it is the peer requirement in current situation, KELM comes out to be best. KELM gives 96.64% accuracy with only 1.599 s of execution time. The techniques performed better as compared to the existing techniques introduced in^21,23,34^. Thus, the researchers and scientists working in the labs can focus on machine learning techniques for CoViD19 detection so that proper treatment can be given to the patients at an early-stage only with faster speed. And in the situation of third wave approaching, this non-iterative machine learning methods can replace the kits utilized these days for the testing.

Also, as various countries are under lockdown during this pandemic, daily life activities are at halt as everything is closed included shopping malls, cinema halls etc. This is resulting in an increase in anxiety level among people, mainly young ones, which further can lead to anger, isolation, mood swings, panic attacks, fear, depression etc. This behavior change is evident on social networking sites like twitter^71^ where the usage of words like bored, frustrated, want to get out etc. has increased after lockdown^72^. Such words depict the mental status of an individual and reports state that such mental status leads to suicidal tendencies. Hence, emotion detection can also be possible with the help of machine learning. By detecting such words of emotions on the social networking sites, it can be made possible to give any individual appropriate counseling at the right time in this serious pandemic.

### Ethics approval

All methods were carried out in accordance with relevant guidelines and regulations.
